# Around the Hospitals

**Published:** 1894-06-02

**Authors:** 


### Around the Hospitals.

[Notice to the Managers of Institutions.—Special space is now reserved for the insertion of notices of hospital meetings and festivals. The Editor requests that all notices may reach The Hospital Office, 428, Strand, at least one week before the date of each meeting.]

**The Sussex County Hospital.**—The accommodation at this institution has now been considerably extended, and the hospital now contains 187 beds. Additional room for female patients has been secured, and a new isolation block has been erected. The upper floor of the Jubilee building has been appropriated to children, so that considerable changes have been effected. The cost of fitting and furnishing the new women's ward, which includes an entirely new oak floor, has been defrayed by a lady, who prefers to remain anonymous. Several other wards require reflooring, a matter which is being considered by the committee, who have to face an extra annual expenditure of over £700, resulting from the extension of the hospital. The expenditure for 1893 was £11,124, extraordinary expenses not included, the income, exclusive of legacies, amounting to £7,988. The munificent legacy of £30,000, resulting from the will of the Rev. J. Spurrell, was invested according to the directions. The number of in-patients treated amounted to 1,522, the out-patients numbering 9,054. The average cost per in-patient was £5 17s. 9d., and of each bed occupied £66 18s.

**St. Mary's Hospital, Paddington.**—Few institutions make a more determined effort to carry out the large amount of work required of them than St. Mary's. The hospital is the only means of gaining skilled care and medical assistance possessed by an immensely widespread district, a district which contains so many well-to-do, that the care of the sick poor should be amply provided for without any special effort. Yet this is not so. The reliable income amounts to about £15,700; the expenditure to £23,000. For many years sufficiently large legacies have fallen to the lot of the hospital, and with the help of these the expenditure has been provided for. Last year the receipt of legacies was so small, that over £7,000 was wanting to defray expenses. The management are unceasing in their efforts to help by curtailing all unnecessary expense, and although the work accomplished was in excess of any previous year, the expenditure was actually reduced by over £300. Each occupied bed cost £4 10s. less, and each patient 13s. less—a most creditable result. It is hoped that the Clarence Memorial Wing will soon be commenced, as the plans are approaching completion. The sum of £25,000 is required to complete the out-patients' department, and towards this £12,582 has been subscribed.

**Devonshire Hospital and Buxton Bath Charity.**—This hospital is intended for the reception of those suffering from such forms of disease as are benefited by the Buxton air and waters, and especially for the treatment of rheumatism. A large number of patients leave wonderfully relieved of their ailments, and last year no less than 2,751 patients resided within the walls of the hospital. Although the original intention of the charity is adhered to, severe cases of accident which would be harmed by want of proper attention or a long journey are not refused admission. During 1893 twenty such cases were received. The hospital is well su ported, and tokens of sympathy and interest from its many friends take useful and practical form. It has been found how much appreciated are the appliances called walking machines, and three have now been presented to the hospital. In all manner of ways the patients are remembered, and every encouragement given to inspire the management of this useful institution with zeal and a confidence that their endeavours are appreciated.

